# Effects of the Pars Plana Vitrectomy on the Chronic Total Rhegmatogenous Retinal Detachment in the Young Adults

**DOI:** 10.3389/fmed.2021.755389

**Published:** 2021-10-22

**Authors:** Jinguo Yu, Xingxing Hu, Jiangkai Zhang, Han Han, Bo Huang, Rodrigo Brant, Cheng Zhang, Hua Yan

**Affiliations:** ^1^Department of Ophthalmology, Tianjin Medical University General Hospital, Tianjin, China; ^2^Laboratory of Molecular Ophthalmology, Tianjin Medical University, Tianjin, China; ^3^Department of Ophthalmology, University of Mississippi Medical Center, Jackson, MS, United States; ^4^Department of Ophthalmology and Visual Sciences, Federal University of São Paulo, São Paulo, Brazil; ^5^Department of Ophthalmology and Visual Sciences, Montefiore Medical Center, Albert Einstein College of Medicine, Bronx, NY, United States

**Keywords:** rhegmatogenous retinal detachment, 23-gauge pars plana vitrectomy, proliferative vitreoretinopathy, young adults, surgery

## Abstract

**Objective:** To observe the characteristics and evaluate the efficacy and safety of the chronic total rhegmatogenous retinal detachment (RRD) treatment by the 23-gauge pars plana vitrectomy (PPV) in young adults and to analyze the related factors.

**Methods:** A retrospective chart review was performed for the young adults who underwent the 23-gauge PPV for the chronic total RRD at the Tianjin Medical University General Hospital from 2011 to 2018. A total of 54 eyes of 48 patients were included in this study. The preoperative vision ranged from 2.00 to 1.00. The mean duration of RRD was 9 ± 0.6 months with a range from 4 to 18 months. The proliferative vitreoretinopathy (PVR) grade D1 and grade D2 was diagnosed in 48 eyes and 6 eyes, respectively. About 37 eyes were filled with C3F8 and 17 eyes were filled with silicone oil tamponade. The follow-up ranged from 9 to 78 months with a mean of 23 ± 2.2 months.

**Results:** The postoperative visual acuity increased in all the eyes at the final observation. The retinal attachment was achieved in 49 eyes (90.7%) in the primary PPV. Five eyes (9.3%) with the failed retinal attachment finally achieved the attachment after the second procedure. The postoperative complications mainly included temporary intraocular pressure (IOP) elevation, hyphema, and retinal redetachment.

**Conclusion:** Chronic total RRD can be treated via the 23-gauge PPV with a great anatomical and visual prognosis in the young adult. The successful treatment of the chronic total RRD in young adults is mainly associated with the complete dissection of the severe vitreoretinopathy, especially for the epiretinal membrane at the retinal breaks and degenerations and the subretinal proliferation during surgery.

## Introduction

Rhegmatogenous retinal detachment (RRD), which often causes difficult recovery of the retinal function due to the delayed retinal reattachment by a surgical procedure, is one of the most common reasons for severely decreased vision in young adults. RRD has multiple etiologies in young adults (1). The characteristics and outcomes of the pediatric RRD have been reported in numerous studies (2–6). It has been reported that the incidence of RRD in the young adults is different from RRD in the adults; it is ~0.38–0.69 per 100,000 individuals in the children and 7.98–12.4 per 100,000 individuals in the adults (2, 7).

Chronic total RRD is one of the most refractory retinal detachments with poor outcomes in young adults. Two approaches, a scleral buckling procedure and pars plana vitrectomy (PPV), are typically used to treat RRD in young adults. The selection of surgical procedures according to the vitreoretinopathy is important for the final outcomes in the chronic total RRD in the young adults. The purpose of choosing an optimal approach to treating the chronic total RRD in young adults is to provide the patients with an individualized management conceptual design according to the etiology and severity of the proliferative vitreoretinopathy (PVR).

The scleral buckling procedure has been an effective surgical approach for RRD for more than 60 years (8). However, this procedure is only compatible with simple RRD. PPV has been adopted in treating RRD with PVR grade C or worse with the successful results in many instances in adults. Since PPV is effective for RRD, this study strictly choose the surgery for the chronic total RRD in young adults because of the difficulty in completely removing the cortical vitreous, higher postoperative intraocular cellular activity, unpredictable tamponade such as silicone oil or gas intraoperatively, and difficulty in maintaining the head position.

In this retrospective study, the anatomical characteristics of the chronic total RRD in the young adults were reported and emphasized the experiences in surgical expertise with the management of the epiretinal membrane around the retinal breaks and retinal degenerations and the subretinal proliferation in treating the chronic total RRD by the 23-gauge PPV.

## Methods

### Patients

This study was approved by the Medical Ethics Committee of the Tianjin Medical University General Hospital and complies with the Declaration of Helsinki including current revisions and with the Good Clinical Practice guidelines. The procedures followed were in accordance with the institutional guidelines and all the subjects provided their written informed consent for the treatment according to the Declaration of Helsinki. All the subjects were recruited from the ophthalmology department of the Tianjin Medical University General Hospital.

In this study, the age of the young adults ranged from 12 to 35 years old. About 54 eyes of 48 consecutive young adult patients who had the chronic total RRD underwent the 23-gauge PPV combined with tamponades of C3F8 or silicone oil at the Tianjin Medical University General Hospital from 2011 to 2018. About 34 patients were male and 14 patients were female. The age ranged from 12 to 33 years with a mean of 23 ± 2 years. The mean refraction of myopia was −6.50 ± −1.25 diopters with a range from −2.75 to −10 diopters. The lens was transparent in all the patients. The mean duration of RRD was 9 ± 0.6 months with a range from 4 to 18 months. The preoperative vision ranged from 2.00 to 1.00 and the mean preoperative intraocular pressure (IOP) was 14.12 ± 2.22 mm Hg. The follow-up ranged from 9 to 78 months with an average of 23 ± 2.2 months (shown in Table 1). Eyes with a prior history of congenital or developmental structural ocular abnormalities, ocular trauma, previous ophthalmologic surgery, retinal laser photocoagulation, ocular tumors, corneal opacity, and preceding uveitis were excluded.

**Table 1:** Characteristics of the young adult patients with chronic total RRD.

**Characteristics**	**Value**
Patients	48
Male	34
Female	14
Eyes	54
Age (years), Mean ± SD (range)	23 ± 2 (12–33)
Myopia (diopters), Mean ± SD (range)	−6.50 ±−1.25 (−2.75 to −10.00)
Duration of RRD (months), Mean ± SD (range)	9 ± 0.6 (4–18)
**PVR classification (eyes, %)**	
Grade D1	48 (88.9)
Grade D2	6 (11.1)
**Number of retinal breaks**, ***n*** **(%)**	
One	16 (29.6)
Two	11 (20.4)
Three	15 (27.8)
Four	6 (11.1)
Six	4 (7.4)
Seven	2 (3.7)
**Site of retinal breaks**, ***n*** **(%)**	
Superior site	16 (29.6)
Inferior site	28 (51.9)
Both sites	10 (18.5)
**Numbers of patients with symptom of visual field defects**	
preop.	49
postop.	0
**BCVA (range)**	
preop.	2.00–1.00
postop.	1.30–0.10
**IOP (mmHg), Mean** **±** **SD**	
preop.	14.12 ± 2.22
postop.	13.75 ± 3.13
Retinal attachment after single surgery, *n* (%)	49 (90.7%)
**Postoperative complications**, ***n*** **(%)**	
Temporary IOP elevation	5 (9.3)
Hyphema	1 (1.9)
Retinal redetachment	5 (9.3)
Follow-up (months), Mean ± SD	23 ± 2.2 (9–78)

*RRD, rhegmatogenous retinal detachment; PVR, proliferative vitreoretinopathy; BCVA, best corrected visual acuity; IOP, intraocular pressure*.

### Preoperative Examinations

Complete preoperative evaluations including symptoms, best corrected visual acuity (BCVA) [logarithm of the minimum angle of resolution (LogMAR)], IOP, duration of retinal detachment, ocular and systemic disease history, and funduscopy were obtained. The extension of retinal detachment, number and location of the retinal break(s), and grade of PVR were evaluated by using the indirect ophthalmoscopy and the Goldmann three-mirror contact lens. Additional B-scan examinations were adopted in all the patients to exclude the other retinal and choroidal diseases.

### Surgical Procedures

All the surgeries were performed by the same surgeon, Dr. Yan, with the 23-gauge PPV by using a three-port technique. The eye received 2% lidocaine retrobulbar anesthesia and was then prepared for a standard three-port 23-gauge PPV. The 23-gauge infusion cannula was placed 3.5 mm behind the limbus at the inferotemporal quadrant; then, the 23-gauge sclerotomies for the optic fibers and vitrectomy (Stellaris® PC, Bausch & Lomb, USA) were created. A non-contact wide-angle viewing system (Resight 700, Carl Zeiss Meditec AG, Jena, Germany) was activated and the fundus could be viewed thoroughly and clearly by the illumination of the light probe. A full examination of the fundus was performed to locate all the retinal breaks and to detect the additional areas of the vitreoretinopathy intraoperatively.

During PPV, the posterior hyaloid separation was induced and completely excised by the suction and cutting by using a vitreous cutter over the optic nerve head in the eyes without the posterior vitreous detachment. Then, triamcinolone (TA) was injected into the vitreous cavity for staining the residual proliferative vitreous and epiretinal membrane that was difficult to discriminate. In each case, the meticulous shaving of the vitreous base and epiretinal membrane at the retinal breaks and degeneration area was performed under a wide-angle viewing system with the assisted sclera depression. Retinectomy was conducted and the subretinal membrane was removed appropriately in the cases with the local severe retinal stiffness and shrinkage. The intraocular laser photocoagulation was applied around the margins of the retinal breaks and degenerations in all the patients without any additional cryotherapy after the air-fluid exchange. About 37 eyes were filled with C3F8 and 17 eyes were filled with silicone oil tamponade. The routine postoperative examinations were performed 1, 2, 3, and 7 days; 2, 3, and 12 weeks; and 1 year after the surgery. The postoperative evaluations included BCVA, IOP, retinal anatomy, and complications. The paired Student's *t*-test was used to analyze the changes in the pre- and postoperative IOP.

## Results

### Best Corrected Visual Acuity

The postoperative BCVA ranged from 1.30 to 0.10 (LogMAR). The BCVA increased in all eyes (100%) at the final follow-up. The symptoms of the visual field defects disappeared in all the eyes (shown in Table 1).

### Retinal Anatomy

#### Retinal Breaks

**Figure 1:**
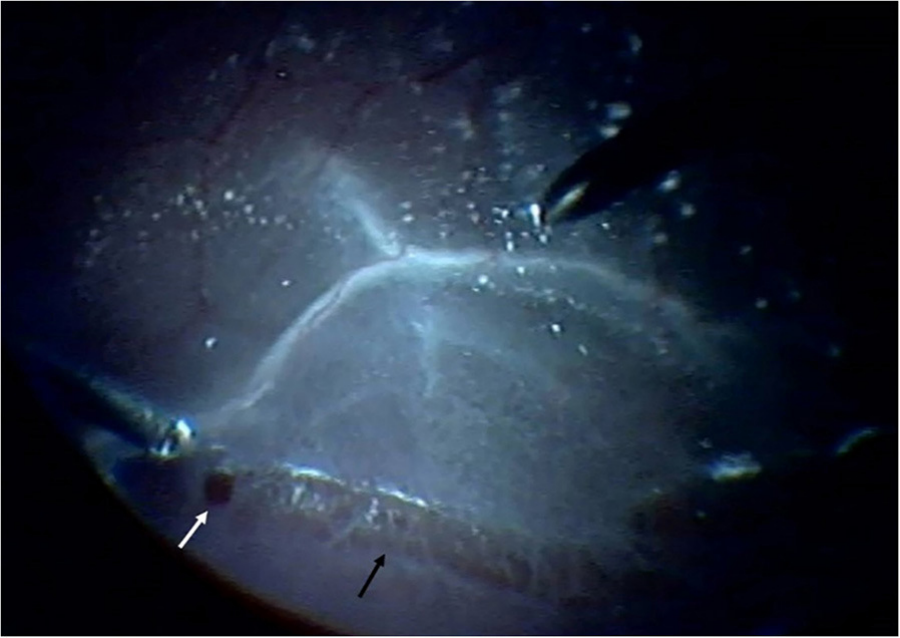
Retinal break (white arrow) and degeneration (black arrow) were observed in the inferior site intraoperatively.

There was only one retinal break in 16 eyes (29.6%), two retinal breaks in 11 eyes (20.4%), three retinal breaks in 15 eyes (27.8%), four retinal breaks in 6 eyes (11.1%), six retinal breaks in 4 eyes (7.4%), and seven retinal breaks in 2 eyes (3.7%). The retinal breaks were found in the superior site in 16 eyes (29.6%), in the inferior site in 28 eyes (51.9%) (shown in Figure 1), and in both the superior and inferior sites in 10 eyes (18.5%) (shown in Table 1).

#### Proliferative Vitreoretinopathy

The criteria of the PVR classification were obtained from the Retina Society Terminology Committee. PVR grade D1 was diagnosed in 48 eyes (88.9%) and PVR grade D2 was diagnosed in 6 eyes (11.1%) (shown in Table 1).

#### Retinal Attachment

**Figure 2:**
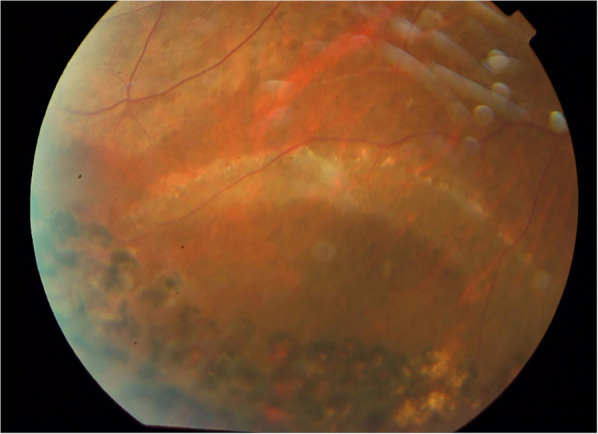
Retinal break closure and retinal attachment were present with the laser scar formation at the final follow-up.

Overall, retinal attachment with a single surgery was achieved in 49 eyes (90.7%) among all the operated eyes (shown in Figure 2). Only five eyes (9.3%) received a second vitrectomy procedure for the recurrent retinal detachment repair and the retina finally attached (100%). The retinal reattachment rate was 100% at the final follow-up.

#### Intraocular Pressure

The postoperative IOP was 13.75 ± 3.13 mm Hg and there was no significant difference between the postoperative and preoperative IOP at the final follow-up (*p* > 0.05).

### Postoperative Complications

The postoperative complications mainly included the temporary IOP elevation in five eyes (9.3%), hyphema in one eye (1.9%), and retinal redetachment in five eyes (9.3%) after the primary PPV. No epiretinal membrane formation occurred after PPV at the final observation.

## Discussion

According to the previous studies, the main reasons for RRD in children are trauma, myopia, previous ophthalmic surgeries, and congenital or developmental anomalies (2, 9–13). However, the idiopathic or unknown causes of retinal detachment are still a common phenomenon in young adults. The retinal breaks are present in 5–10% of the general public; however, very few lead to RRD. PVR is always significant when RRD is diagnosed in young adults. In this study, grade D PVR was present in all the young adult RRD patients, possibly because of their delayed diagnosis. Akabane (14) reported a PVR incidence in the children was 29.8–37.5%, while in adults, it was 5–10%. Therefore, the early precision examination, diagnosis, and treatment are important in young adults who have the symptoms of blurred vision or vision loss and potential RRD.

The symptoms and retinal structures of the chronic total RRD in young adults have their own special characteristics. Young adults with RRD may not have any complaints of the decreased vision or loss of vision at the beginning and a few patients detect the decreased vision incidentally when covering the fellow eye due to the chronic and mild retinal detachment. In this study, the most common presenting symptom was a gradual vision decrease or loss of vision in 88% of the eyes and the retinal detachments had a longer history ranging from 4 to 18 months. Sultan (15) reported that the most common presenting symptom was a gradual vision decrease or loss of vision in 45.7% of the eyes and the incidental findings in the routine examinations or follow-up in 23% of the eyes. Gonzales (13) reported that 46% of the patients had to present symptoms related to retinal detachment. There is no obvious ocular history in most of the RRD in young adults; therefore, complete and careful examinations of the vitreous and retina should be performed by the different methods preoperatively.

Since all the retinal breaks were small and/or various in the patients, this study detected all the retinal breaks by the indirect ophthalmoscopy and the Goldmann three-mirror contact lens preoperatively. In this study, total retinal detachment was found in all the eyes and the main reasons might be the small retinal holes and chronic nature. Sultan (15) reported that the vast majority of the cases had retinal breaks or holes identified in 86.75% of the eyes intraoperatively, while no retinal breaks or holes were identified in 7.22% of the eyes. In this study, it is found that all the retinal breaks, including the atrophic holes in the lattice, were the round holes with RRD and without any horseshoe tears. There was only one retinal hole in 29.6% of the eyes, two retinal holes in 20.4% of the eyes, and multiple holes in 50% of the eyes. The retinal breaks were found in the superior site in 29.6% of the eyes, in the inferior site in 51.9% of the eyes, and in both the superior and inferior sites in 18.5% of the eyes. Sultan (15) reported multiple holes in 22.3% of the eyes with RRD in the children. In this study, there was no macular hole, giant retinal tear, or retinal dialysis. Sultan (15) reported that 20.8% of the eyes with RRD had retinal dialysis and giant tears were found in 22.9% of the eyes. Yokoyama (3) reported retinal dialysis in 27% of RRD in the children. From the previous study, it is known that after an RRD in one eye, the risk of RRD is ~10% in the fellow eye. Therefore, the fellow eye should be examined carefully and treated early.

The surgical repair methods in the young adults RRD generally include the buckling procedure and PPV. The selection of surgical repair for the young adults RRD depends on the etiology and features of retinal detachment such as the severity of PVR, site, size, and the number of the retinal breaks. The scleral buckling procedure was the commonly used method for the treatment of RRD in young adults before PPV was widely used (16). However, the scleral buckling or band as the primary procedure was not commonly used in the total RRD after the PPV era. In this study, all the RRDs had a chronic nature and PVR was severe at grade D. Total RRD in the young adults with PVR grade D cannot be treated successfully with the scleral buckling procedure. Therefore, the only vitrectomy is the optimal method for treating the chronic total RRD with PVR grade D in young adults by completely eliminating PVR, peeling the epiretinal membrane, and even performing retinectomy for the removal of the subretinal membrane. Some surgeons reported that the vitrectomy with or without a buckling procedure was adopted in the primary surgical procedure for RRD in the children and achieved better results (14, 15, 17–19).

Treatment of the chronic total RRD with the 23-gauge PPV in the young adults has superiority with more safety, quick recovery, and less damage compared to the 20-gauge PPV due to a small sutureless self-sealing incision. The technique of treating the chronic total RRD in young adults is different from the fresh RRD for its own peculiarity of vitreoretinopathy, especially at the retinal holes and degenerations. Performing complete removal of the cortical vitreous on the detached retina is often difficult, as there is less frequent posterior vitreous detachment and stronger vitreoretinal adhesion in the young adult patients with RRD. The most important procedure of treating the chronic total RRD in young adults during vitrectomy is to address the vitreous adherent to the retinal holes and degenerations. Usually, the vitreous is ropier in young adults, especially at the vitreous base and peripheral retinal area (10, 20). In addition, the posterior vitreous adherent to the epiretinal proliferative membrane becomes tighter at the retinal holes and degeneration, which is more difficult to excise completely during the vitrectomy. In this study, five eyes with the failed retinal attachment at the primary PPV had the retinal detachment at the inferior site and the epiretinal membrane and vitreous traction were not completely removed. Therefore, in cases with the retinal detachment at the inferior site, the local proliferative vitreous and epiretinal membrane should be excised completely because they can cause recurrent retinal detachment, which is easily induced by the traction of the residual vitreous and epiretinal membrane. Else, the successful retinal attachment will not be obtained even with C3F8 or silicone oil tamponade. Conversely, the scleral buckling procedure may have a better success rate in the retinal attachment because it does not disturb the intraocular tissue compared with vitrectomy. TA is always used for staining the proliferative vitreous and epiretinal membrane during the vitrectomy (21), but with TA, difficulty in discriminating the proliferative vitreous and epiretinal membrane can be easily resolved, providing the clear observation and thorough excision that promote the final successful rate by closing the retinal hole with the laser photocoagulation during surgery. Both the cryopexy and laserpexy can be used for closing the retinal holes and as the prophylactic treatment for retinal degenerations. Generally, the vitreous and retina have a proliferative trend after the cryotherapy for the retinal hole closure and retinal degeneration compared with after laser photocoagulation (22, 23) and this effect is more significant in young adults than in adults. Therefore, laser photocoagulation was adopted to manage the retinal holes and degenerations in all of our patients who did not experience any related postoperative proliferations. Retinectomy is a better option to preserve the retinal attachment in cases with local severe retinal stiffness and shrinkage induced by fibrosis. Adequate retinectomy should be performed and the subretinal membranes should be removed appropriately if needed. Complete subretinal fluid drainage during the vitrectomy seems to be unnecessary for all the RRD surgical procedures in cases with a retinal hole in the peripheral area.

The final visual outcome after the treatment of the chronic total RRD in the young adults is associated with retinal attachment, especially for the macular attachment and function. Since the retinal hole closure and retinal attachment are achieved, the final vision improvement is still limited in some of the cases due to the long-term retinal and macular detachment. In this study, the postoperative vision ranged from 1.30 to 0.10 with increased vision in 100% of the eyes at the final follow-up. The symptoms of the visual field defects disappeared in all the eyes. Since 100% of the eyes achieved the vision improvement after the successful retinal attachment, the vision remained inadequate postoperatively due to the chronic nature of total RRD, initial poor vision, greater extent of RRD such as total RRD, and the presence of preoperative PVR grade D. This analysis of poor postoperative vision is almost the same compared to the previous reports (3, 4, 13, 16, 24).

In this study, only five eyes (9.3%) had postoperative temporary IOP elevation. This temporary elevation is probably caused by the intrinsic characteristics of RRD and/or surgical procedures. In patients with RRD, the aqueous humor circulation has a passageway in addition to the routine anterior chamber passageway in which the aqueous humor outflows and is absorbed through the choroid and sclera due to the retinal detachment that induces decreased IOP. However, the passageway of the aqueous humor outflowing through the choroid and sclera is significantly decreased or stopped due to the closure of the retinal holes and retinal attachment after vitrectomy and the aqueous humor mainly outflows through the anterior chamber intensively. Therefore, IOP elevation may occur in some of the successful retinal attachment cases. Alternatively, an early postoperative IOP increase may be caused by the severe inflammation and ciliary body stimulation during the vitrectomy and the possible mechanisms include ciliary body edema and inflammatory trabecular meshwork obstruction. Other reasons for the postoperative IOP elevation may be intraocular gas expansion, a pupillary block from gas or fibrin, erythroclastic glaucoma, and silicon oil-related glaucoma (25–28).

The risk factors of hyphema after the RRD surgeries include high myopia, aspirin treatment, cryotherapy impacts, trans-scleral drainage, scleral buckling, PPV, surgery duration, IOP, and systemic hypertension (29). In this study, only one eye (1.9%) had hyphema postoperatively and it was absorbed spontaneously within 7 days. The anterior chamber bleeding might come from the sclerotomies rather than from the episcleral vessels. In this study, the occurrence of hyphema did not affect the final anatomical success rate which was the same as compared to the previous studies (15, 30). The single-operation anatomical success rate was 90.7% in the primary PPV and 100% after the second PPV. Sultan (15) reported that the single-operation anatomical success rate was 74.2% in the primary PPV group and 77% in the second PPV group.

In summary, since, the 23-gauge PPV is an effective and safe approach by completely removing the epiretinal proliferation around the retinal breaks and retinal degenerations and the subretinal proliferation under a non-contact wide-angle viewing system in treating the chronic total RRD of the young adults, the vitreoretinal surgery should be prudently employed in treating the fresh RRD because it is more complex compared to the scleral buckling procedures. The details of symptoms and characteristics of the chronic total RRD in young adults should be emphasized.

## Data Availability Statement

The raw data supporting the conclusions of this article will be made available by the authors, without undue reservation.

## Ethics Statement

The studies involving human participants were reviewed and approved by Tianjin Medical University General Hospital Medical Ethics Committee. Written informed consent to participate in this study was provided by the participants' legal guardian/next of kin.

## Author Contributions

HY and JY: research design and manuscript preparation. JY and XH: data acquisition, research design, and data analysis and manuscript preparation. JZ and HH: data acquisition and data analysis and manuscript preparation. BH, RB, and CZ: research design and manuscript preparation. All authors contributed to the article and approved the submitted version.

## Funding

This is supported by the National Natural Science Foundation of China (Grant Number 82020108007, 81830026), the Beijing-Tianjin-Hebei Special Project (Grant Number 19JCZDJC64300(Z), 20JCZXJC00180), and the Tianjin Natural Science Foundation (Grant Number 19JCQNJC11300).

## Conflict of Interest

The authors declare that the research was conducted in the absence of any commercial or financial relationships that could be construed as a potential conflict of interest.

## Publisher's Note

All claims expressed in this article are solely those of the authors and do not necessarily represent those of their affiliated organizations, or those of the publisher, the editors and the reviewers. Any product that may be evaluated in this article, or claim that may be made by its manufacturer, is not guaranteed or endorsed by the publisher.
